# Prevalence and risk factors of musculoskeletal injuries in modern and contemporary dancers: a systematic review and meta-analysis

**DOI:** 10.3389/fpubh.2024.1325536

**Published:** 2024-02-28

**Authors:** Yufei Sun, Hui Liu

**Affiliations:** ^1^College of Human Sport Science of Beijing Sport University, Beijing, China; ^2^China Sports Health Research Institute of Beijing Sport University, Beijing, China

**Keywords:** meta-analysis, dance injury, musculoskeletal injury, modern dance, contemporary dance

## Abstract

**Background:**

A large number of studies have found that the musculoskeletal injury of modern and contemporary dancers has a high incidence. Previous publications have indicated that there are many potential factors that are related to dancing injury; however, they have not been proven, and even some data are conflicting in different research.

**Results:**

The search yielded 18 prospective studies reporting on professional and pre-professional modern or contemporary dancers from companies and schools. The prevalence of modern and contemporary dancers was 0.82 (*95% CI*: 0.74~0.90). The injury proportion of trauma, overuse, ankle and foot, lower extremity, joint and ligaments, muscle and tendons, and time-loss were 0.40, 0.26, 0.49, 0.34, 0.33, and 0.29 in the total number of injuries. There was no evidence of a significant difference in sex, age, and education program. The factors of BMI and injury history achieved statistical significance, and the *p*-values were less than 0.01.

**Conclusion:**

Based on the results of this article, BMI and injury history may be risk factors for injury in contemporary and modern dancers. Modern and contemporary dancers have a high prevalence of musculoskeletal injuries. Lower extremity injuries are the most common in the whole body, with injuries to the foot and ankle being more frequent. The mechanism of injury is mostly overuse injury, and the injured tissues are mostly muscle tendons and joint ligaments.

## Introduction

Injuries are common among dancers and can result in an inability to attend classes, rehearsals, or performances, or even an early end to dancing (1). Some dancers will suffer from osteoarthritis, lumbar disc herniation, and other diseases after retirement. These kinds of diseases could result in lameness and walking difficulties, which greatly affect their quality of life (2). From 2014 to 2018, 4,152 patients were admitted to emergency departments for dance injuries in the United States (3). The musculoskeletal injury prevalence of ballet dancers is more than 80%, especially in the lower limbs such as the ankle and knee (4). The prevalence of other dances, such as modern dance and contemporary dance, is also higher than 60% (5). The University of the Arts Rotterdam in the Netherlands has conducted a number of prospective studies on undergraduate students majoring in contemporary dance from various perspectives, such as psychology, training load, and personal conditions. The results show that the risk factors of injury are related to stress (a visual analog scale is used to measure stress scores), sex, BMI, injury history, and other factors. Meanwhile, the injury sites are mostly concentrated in the ankle joint and foot (6–9). There are also many research studies (10–13) on professional dancers in modern and contemporary dance companies. Injuries to professional dancers can add to the financial expenses of dance companies, such as medical compensation and treatment of injuries. One of the most significant factors that lead to injuries is high dynamic loads of an impact nature that occur particularly during the landing phase of numerous jumps in modern dance (14). At the landing phase, the joints of low limbs sustain high values of the vertical component of ground reaction force, which may reach 7.4 BW (15–17). A research study used the session rating of perceived exertion (sRPE) method to calculate the training load (TL) of contemporary dancers and found that the average weekly TL was 6,685 ± 1,605 arbitrary units (18).

Existing studies have shown that the injury of modern and contemporary dancers has a high prevalence and many potential risk factors, but current research cannot determine the mechanism of injury caused by each factor. The differences in choreography techniques or training modes will also lead to different injury conditions for dancers (9). Some risk factors have not been confirmed in other studies, and some studies have found the opposite for some factors, such as BMI and gender (2, 8). However, compared with the maturity of ballet research, modern and contemporary dance has a wide range of contents and themes (19). Some colleges or dance companies of modern and contemporary dance at home and abroad have not yet formed a unified art school or training system (13). Therefore, identifying the common factors and mechanisms of injury in modern and contemporary dancers will be helpful for further scientific and extensive injury prevention and rehabilitation work. This study systematically collected high-quality prospective studies on the musculoskeletal prevalence of modern and contemporary dancers and its risk factors. After comprehensively and quantitatively evaluating the relationship between various risk factors and dance injury by means of meta-analysis, it proposed scientific basis and intervention measures for the prevention of dance injury.

## Methods

### Search strategy and selection criteria

Using PubMed and Web of Science electronic databases, we searched for prospective studies that included the epidemiological investigation of contemporary dance injury. The two bibliographical databases were screened for eligible studies on 20 June 2023. The researchers deleted repeated articles using the following keywords: “dance,” “dancing,” “modern dance,” “injuries,” “wounds,” “trauma,” “wounds and injuries,” “prospective study.” The search strategy was based on different combinations of words:((dance[tw] OR “Dancing”[Mesh] OR “modern dance”[tw]) AND (injuries*[tw] OR wounds*[tw] OR trauma[tw] OR “Wounds and Injuries”[Mesh])) AND (“prospective study”[tw] OR “prospective studies*”[tw]).

Original prospective cohort studies published in English, reporting on dance prevalence and risk factors, objecting to pre-professional and professional contemporary dancers, and using the same definitions of injury and injury risk factors were included in the present meta-analysis. The reference lists of all reviewed articles were subsequently hand-searched for potentially eligible studies.

The exclusion criteria were: (1) research participants are not modern or contemporary dancers, (2) unsuitable study design, (3) injury type is not musculoskeletal injury, (4) information required for this study was not reported, such as the different risk factors, and (5) use of the same participants in different articles.

### Data extraction

Data from the included studies were extracted and tabulated using a pre-piloted form for synthesis and study quality assessment. All data were extracted independently by two reviewers (S.Y.F. and L.X.X.) and potential discrepancies were resolved through discussions with a third author (L.H.). We collected the following data for each study which included study characteristics (title, first author name, source, study design, year of publication, and region of study), study participants, number of injured participants, number of injuries, injury definition, and definition of different risk factors.

### Sensitivity analyses and meta-analysis

Categorical variables were summarized using frequencies and percentages, while continuous variables were summarized as means and standard deviations (SD). Odds ratios (OR) and 95% confidence intervals (CI) were used for categorical outcomes. The weighted mean difference (WMD) and its 95% CI were calculated for continuous outcomes. Between-study heterogeneity assessment was conducted using the Cochran *Q* statistic and *I^2^*. High heterogeneity was confirmed with a significance level of *p* < 0.05 and *I^2^* > 50%. The random effects model (DerSimonian-Larid) was utilized to calculate pooled effect estimates and high heterogeneity was encountered. The fixed effects model (Mantel–Haenszel) was used for comparison with low heterogeneity. Sensitivity analyses were performed by excluding studies one by one to determine whether our results changed. If heterogeneity decreased significantly, it indicated that the literature was one of the main sources of heterogeneity. Heterogeneous risk factors were analyzed by subgroup analysis and meta-regression according to the year of publication and the grade of the participants. If the heterogeneity test was *p* > 0.05 and effect estimation was *p* < 0.05, it indicated that the source of heterogeneity can be explained. Statistical significance was set at *p* < 0.05 for all comparisons and all *p*-values were two-sided. Review Manager software, Version 5.3 (RevMan, 2014, The Nordic Cochrane Center, The Cochrane Collaboration, Copenhagen, Demark) was used to conduct a meta-analysis of the risk factors of injury, and STATA IC15 (Stata LLC, College Station, Texas) was used to conduct a meta-analysis of prevalence and proportion of different types of injury. The meta-analysis was performed according to the Preferred Reporting Items for Systematic Reviews and Meta-Analyses (PRISMA) guidelines.

### Study quality and publication bias assessment

In this study, the Newcastle-Ottawa Scale (NOS) was used to evaluate the quality of the included literature. NOS is a commonly used quality evaluation tool for meta-analysis, which is evaluated through three modules with a total of eight entries. Specifically, it includes selection, comparability, exposure, and results. NOS evaluates the quality of the literature using the semi-quantitative principle of the scoring system, except for comparability, which can be evaluated with a maximum of two points; the rest of the entries can be evaluated with a maximum of one point and a total score of nine, with the higher score suggesting the higher quality of the study (with a score of ≥6 denoting high quality).

Publication bias was evaluated by funnel plot, Egger regression, and Begg rank correlation. If *p* > 0.05, it indicates no publication bias; otherwise, it is considered to have publication bias.

### Patient and public involvement

No patients were involved in this study.

## Results

### Article selection and injury demographics

The online literature search identified 95 potentially relevant records. After duplicate removal, 83 titles and abstracts were reviewed. Of these, 65 articles were deemed ineligible and excluded. Eighteen studies were selected for full-text review and all were included in the meta-analysis. However, the articles reported different data required for this study, and there were issues with different articles being the same group of participants; articles that were not the same were included in each analysis. There were 13 articles included in the analysis of prevalence, 12 articles included in the injury proportion, and 6 articles included in the risk factors, as well as 7 articles included in trauma injury, 8 articles included in overuse injury, 10 articles included in ankle and foot injury, 5 articles included in lower extremity injury, 6 articles included in the joint and ligaments injury, 6 articles included in muscle and tendons injury, and 7 articles included in time-loss injury (TL-inj) (Table 1).

**Table 1:** The flow diagram of meta-analysis.

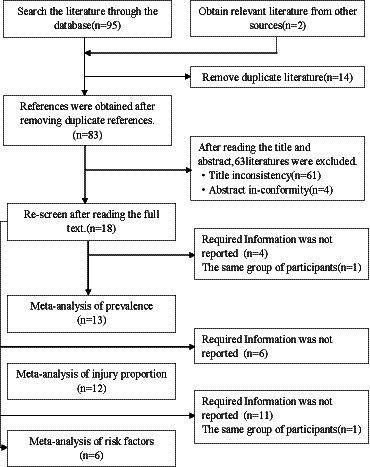	

### Quality assessment of included studies

Two reviewers (S.Y.F. and L.X.X.) independently rated the quality of eligible studies and a synthesis of their reports was performed. The mean NOS score for the 18 prospective cohort studies was 7.8 ± 1.1(three papers scored 9 points, eight papers scored 8 points, and seven papers scored 7 points, all of which were of high quality).

#### Prevalence

The overall OR of injury was 0.82 (95% *CI*: 0.74~0.90). Subgroup analysis was performed on the analysis results. Subgroup analysis of study years was conducted: OR for the 1 year group was 0.77 (95% *CI*: 0.59~0.91), OR for the greater than 1 year group was 0.79 (95% *CI*: 0.57~0.94), and OR for the less than 1 year group was 0.89 (95% *CI*: 0.75~0.98). Subgroup analysis of dancer types was also conducted: OR for professional dancers was 0.76 (95% *CI*: 0.62~0.87), while for pre-professional dancers was 0.87 (95% *CI*: 0.76~0.96). The random effects model was used to test the heterogeneity, and the results were *p* > 0.05 and *I^2^* = 93.4%, indicating significant heterogeneity among the included references. In the sensitivity analysis, there was no difference between the OR and *95% CI* after removing each paper one by one and switching the fixed effects model and random effects model, and the result was relatively stable. In the publication bias detection, the value of p of the Egger test was 0.63 > 0.05, so there was no publication bias.

#### Trauma

The overall OR of trauma injury was 0.40 (95% *CI*: 0.20~0.61). Subgroup analysis was performed on the analysis results. Subgroup analysis of study years was conducted: OR for the 1 year group was 0.47 (95% *CI*: 0.27~0.68) and OR for the greater than 1 year group was 0.21 (95% *CI*: 0.19~0.22). The random effects model was used to test the heterogeneity, and the results were *p* < 0.05 and *I^2^* = 99.5%, indicating significant heterogeneity among the included references. In the sensitivity analysis, there was no difference between the OR and *95% CI* after removing each paper one by one and switching the fixed effects model and random effects model, and the result was relatively stable. In the publication bias detection, the value of p of the Egger test was 0.88 > 0.05, so there was no publication bias.

#### Overuse

The overall OR of injury was 0.49 (95% *CI*: 0.28~0.71). Subgroup analysis was performed on the analysis results. Subgroup analysis of study years was conducted: OR for the 1 year group was 0.51 (95% *CI*: 0.33~0.69), OR for the greater than 1 year group was 0.68 (95% *CI*: 0.66~0.69), and OR for the less than 1 year group was 0.20 (95% *CI*: 0.15~0.27). Subgroup analysis of dancer types was also conducted: OR for professional dancers was 0.47 (95% *CI*: 0.34~0.60), while for pre-professional dancers was 0.81 (95% *CI*: 0.79~0.82). The random effects model was used to test the heterogeneity, and the results were *p* < 0.05 and *I^2^* = 99.5%, indicating significant heterogeneity among the included references. In the sensitivity analysis, there was no difference between the OR and *95% CI* after removing each paper one by one and switching the fixed effects model and random effects model, and the result was relatively stable. In the publication bias detection, the value of *p* of the Egger test was 0.70 > 0.05, so there was no publication bias.

#### Ankle and foot

The overall OR of injury was 0.26 (95% *CI*: 0.19~0.34). Subgroup analysis was performed on the analysis results. Subgroup analysis of study years was conducted: OR for the 1 year group was 0.29 (95% *CI*: 0.13~0.48), OR for the greater than 1 year group was 0.22 (95% *CI*: 0.20~0.24), and OR for the less than 1 year group was 0.25 (95% *CI*: 0.17~0.33). Subgroup analysis of dancer types was also conducted: OR for professional dancers was 0.29 (95% *CI*: 0.13~0.48), while for pre-professional dancers was 0.24 (95% *CI*: 0.18~0.30). The random effects model was used to test the heterogeneity, and the results were *p* > 0.05 and *I^2^* = 97.1%, indicating significant heterogeneity among the included references. In the sensitivity analysis, there was no difference between the OR and *95% CI* after removing each paper one by one and switching the fixed effects model and random effects model, and the result was relatively stable. In the publication bias detection, the value of *p* of the Egger test was 0.23 > 0.05, so there was no publication bias.

#### Lower extremity

The overall OR of injury was 0.49 (95% *CI*: 0.28~0.71). The random effects model was used to test the heterogeneity, and the results were *I^2^* = 97.6%, indicating significant heterogeneity between the OR and 95% *CI* after removing each paper one by one and switching the fixed effects model and random effects model, and the result was relatively stable. In the publication bias detection, the value of p of the Egger test was 0.79 > 0.05, so there was no publication bias.

#### Joint and ligaments

The overall OR of injury was 0.34 (95% *CI*: 0.23~0.44). The random effects model was used to test the heterogeneity, and the results were *I^2^* = 95.6%, indicating significant heterogeneity between the OR and 95% *CI* after removing each paper one by one and switching the fixed effects model and random effects model, and the result was relatively stable. In the publication bias detection, the value of p of the Egger test was 0.12 > 0.05, so there was no publication bias.

#### Muscle and tendons

The overall OR of injury was 0.33 (95% *CI*: 0.23~0.46). The random effects model was used to test the heterogeneity, and the results were *I^2^* = 96.5%, indicating significant heterogeneity between the OR and 95% *CI* after removing each paper one by one and switching the fixed effects model and random effects model, and the result was relatively stable. In the publication bias detection, the value of *p* of the Egger test was 0.56 > 0.05, so there was no publication bias.

#### Time loss

The overall OR of injury was 0.29 (95% *CI*: 0.17~0.42). Subgroup analysis was performed on the analysis results. Subgroup analysis of study years was conducted: OR for the 1 year group was 0.49 (95% *CI*: 0.46~0.51), OR for the greater than 1 year group was 0.20 (95% *CI*: 0.18~0.23), and OR for the less than 1 year group was 0.25 (95% *CI*: 0.13~0.40). Subgroup analysis of dancer types was also conducted: OR for professional dancers was 0.30 (95% *CI*: 0.08~0.58), while for pre-professional dancers was 0.28 (95% *CI*: 0.11~0.40). The random effects model was used to test the heterogeneity, and the results were *p* > 0.05 and *I^2^* = 98.7%, indicating significant heterogeneity among the included references. In the sensitivity analysis, there was no difference between the OR and *95% CI* after removing each paper one by one and switching the fixed effects model and random effects model, and the result was relatively stable. In the publication bias detection, the value of p of the Egger test was 0.59 > 0.05, so there was no publication bias.

### Risk factors for injury

After the collection and combination of multiple studies, the same risk factors were reported including sex, age, BMI, injury history, and education program which includes bachelor dance (BD) and bachelor dance teacher (BDT). Effect value of the relationship between risk factors and injury: OR of multivariate analysis of variance.

#### Sex

In the study of sex, five papers were included, with a total of 154 male dancers and 424 female dancers. The merge OR and 95% CI of gender factors were 0.97 (0.59~1.60) and *p* = 0.91 > 0.05, respectively, showing no statistical significance. The fixed effect model was used to test the heterogeneity, and the results were *Q* = 5.16, *p* = 0.27, and *I^2^* = 23%, so there was no heterogeneity among the included references. In the publication bias detection, the value of p of the Egger test was 0.34 > 0.05, so there was no publication bias.

#### Age

A total of four papers were included in the study of age, with a total of 231 patients in the injury group, ranging in age from 16.9 to 28.3 years, with an average age of 20.01 years. A total of 208 patients in the uninjured group ranged in age from 17.53 to 30.50 years, with an average age of 20.10 years. The WMD and *95% CI* of age factors were −0.02 (−0.29~0.26) and the value of p was 0.91, respectively, showing no statistical significance. The fixed effect model was used to test the heterogeneity, and the results were *Q* = 4.32(*n* = 4), *p* = 0.23 > 0.05, and *I^2^* = 31%, so there was no heterogeneity among the included references. In the sensitivity analysis, there was no difference between the merge OR and 95% CI after removing each paper one by one and switching the fixed effects model and random effects model, and the result was relatively stable. In the publication bias detection, the value of *p* of the Egger test was 0.52 > 0.05, so there was no publication bias.

#### BMI

In the study on BMI, a total of three papers were included, with a total of 100 patients in the injured group and 271 in the non-injured group. The WMD and *95% CI* of BMI factors were 0.51 (0.11~0.91) and *p* = 0.01, and the difference was statistically significant. The fixed effect model was used to test the heterogeneity, and the results were *Q* = 1.60, *p* = 0.45 > 0.05, and *I^2^* = 0, so there was no heterogeneity among the included references. In the sensitivity analysis, there was no difference between the merge OR and *95% CI* after removing each paper one by one and switching the fixed effects model and random effects model, and the result was relatively stable. In the publication bias detection, the value of p of the Egger test was 0.28 > 0.05, so there was no publication bias.

#### Injury history

Three papers were included in the study of injury history, with a total of 96 people with injury history and 223 people without injury history. The merge ORs of injury history actors and their *95% CI* were 2.25 (1.31~3.87) and *p* = 0.003; the difference was statistically significant. The fixed effect model was used to test the heterogeneity, and the results were *Q* = 1.31, *p* = 0.52 > 0.05, and *I^2^* = 0, so there was no heterogeneity among the included references. In the sensitivity analysis, there was no difference between the merge OR and *95% CI* after removing each paper one by one and switching the fixed effects model and random effects model, and the result was relatively stable. In the publication bias detection, the value of *p* of the Egger test was 0.67 > 0.05, so there was no publication bias.

#### Education program

Three papers were included in the study of the education program, with 200 people majoring in dance performance and 129 people majoring in dance education. The OR and 95% CI of the education program factor were 0.91 (0.58~1.44) and the *p* = 0.70, respectively, showing no statistical significance. The fixed effect model was used to test the heterogeneity, and the results were *Q* = 3.44, *p* = 0.18 > 0.05, and *I^2^* = 42%, so there was no heterogeneity among the included references. In the publication bias detection, the value of p of the Egger test was 0.33 > 0.05, so there was no publication bias.

## Discussion

This meta-analysis studied the prevalence and risk factors of musculoskeletal injury in modern and contemporary dancers. The 18 studies included in this study were published between 2003 and 2021, and the research participants were all modern and contemporary dancers, including students in colleges and professional dancers in companies. Research methods and outcome index measurement methods were specified in the included studies, and their quality met the requirements, so the reliability was high. This research innovatively used meta-analysis to study musculoskeletal injuries in contemporary and modern dance. The results showed that both types of dancers had high rates of injuries, with high BMI and history of injury being significant among the five risk factors included.

The results of this study show that the prevalence of musculoskeletal injury in modern and contemporary dancers is very high, which may be related to the characteristics of performance requirements. Modern and contemporary dance have a high demand for flexibility, control, and technical movements. In a report on the injury mechanism of dancers, 65% of the injuries were related to dance-specific technical movements such as excessive joint flexion and landing imbalance (20). The literature included in this study shows that the annual prevalence of contemporary dancers accounts for 67%. The high prevalence should arouse the vigilance of students and teachers and make them carry out health education or medical treatment and other effective measures for prevention and treatment timely. In addition, the heterogeneity among the included studies was high. After subgroup analysis for the study years, the results showed that the prevalence of dancers in studies with a study period of less than 1 year was significantly higher than the overall prevalence and articles with a study period of more than 1 year. This may be due to the random nature of the time period chosen by the researchers and the instability of the prevalence of dancers over a short period of time, which weakens the effect of this problem when the study period grows. Pre-professional dancers had a higher prevalence than professional dancers when analyzed by subgroup by participant type, which was consistent with the results of previous studies (11). The immaturity of dance technique and the lack of self-protection may be important reasons for the high prevalence in pre-professional dancers.

The results of this study show that the main mechanism of injury in modern and contemporary dancers is overuse rather than trauma. Subgroup analysis of trauma by year of study, one-year studies reported higher numbers of trauma-producing individuals than other studies. It is possible that as the duration of the study increased, a higher percentage of dancers experienced overuse injuries. This was confirmed in a subgroup analysis for overuse injuries, which showed that the proportion gradually increased from 20% to 68%. After a subgroup analysis of overuse by participant type, the proportion of overuse injury is much higher in pre-professional dancers. Students often do not want to miss classes or rehearsals and insist on dancing even when they are not feeling well, lacking a sense of rest and rehabilitation, resulting in the accumulation of injuries. In addition to the early start time of dance training, dance exposure hour is often even more than in competitive sports, which is up to 6 to 8 h a day. The excessive exposure time of dance undoubtedly increases the physical burden of dancers. An epidemiological study (20) shows that the main cause of injury in dance is overuse. When the body function is not recovered enough to withstand the high-load intensity training, great harm will be caused to dancers. At present, the emergency treatment measures for sports and sports rehabilitation physiotherapy methods are relatively mature, which may be related to the purpose of sports competition. However, as an artistic activity, dancers’ investment in the treatment and rehabilitation of diseases has not been paid much attention. This suggests that dance teachers should provide guidance and proper rest to injured dancers, resolutely oppose training with injuries, and encourage dancers to seek medical treatment actively, so as to prevent the aggravation of injuries.

According to the results, lower extremity injuries account for more than half of all body injuries, and among them, foot and ankle injury is the main part of the lower limb injury. By subgroup analysis, the proportion of lower extremity or foot and ankle injuries did not vary significantly between subgroups of different dancer types and years of study, all ranging from 20% to 30%. Similar to ballet (21), dancers’ injuries are all concentrated in the lower extremities, such as the feet and ankles. Modern dancers frequently lack footwear and are required to perform choreography in diverse dance genres (11). Ballet dancers mostly wear pointe shoes that cause ankle sprains. Without the protection of shoes and socks, modern or contemporary dancers need to do tumbling, dance control, and even foot support movements on rubber or wooden floors. However, different dance genres may cause different injury sites. In the present study (13), most modern dancers studied between two and four different styles. Solomon and Micheli (22) conducted a study that compared modern dance techniques (Graham, Horton, and Cunningham), and the Graham technique produced more knee injuries than Cunningham or Horton, while Horton dancers suffered the most back injuries.

Muscles/tendons and joints/ligaments accounted for almost the same proportion of injured tissue (34 and 33%). The high heterogeneity among the included articles may be due to the different definitions of injury. Bronner (23) suggested that half of the time loss injuries were categorized as muscle/tendon tissue but comprised 70% of taking-care-of-business complaints. However, the percentage of these two types of injured tissues was significantly higher than in bone and skin (10). In addition, comparisons between muscles/tendons and joints/ligaments showed differences under the sites of injury. Most joint and ligament injuries involved the lower back, pelvis, and sacrum (27%), while the majority of muscle and tendon tissue injuries mostly involved the hip and groin (17%) (21).

The occurrence of time loss injury to the total number of injuries was common. Due to the high heterogeneity among the included articles, we performed a subgroup analysis of the results. When grouping based on years of studies, the group with a study duration of 1 year had a much higher proportion of time loss injury than the other two groups (more than 1 year or less than 1 year), which showed almost the same proportion. When performing subgroup analysis by dancer type, the results were the same between pre-professional and professional dancers. However, many dancers chose to manage their injuries themselves because of an unwillingness or inability to stop dancing which may cause an underestimation of TL-inj. The impact of a time loss injury can be significant, whether in a dance school or company, because it prevents dancers from working and studying properly which can be detrimental to the school’s teaching schedule and the company’s profitability (24). The ballet company had treated dancers with complaints, perhaps preventing minor issues from becoming TL-inj as reported in ballet dancers (25).

The results of this study suggest that sex is not a risk factor for modern and contemporary dance injury. Male and female dancers have differences in dance movements or training methods, which may cause different causes and natures of injuries. Due to inconsistent results, it is impossible to distinguish the difference in prevalence between male and female dancers. In the research on ankle injury of contemporary dancers, the results of one-way analysis of variance show that the ankle prevalence of male dancers is lower (6). In an earlier study (19), the results showed that the probability of male injury was approximately twice that of female injury. These differences could be caused by the different definitions of injury and the body regions. There is no consensus on whether sex has an effect on the injury of modern dancers.

The results of this study suggest that age is not a risk factor for injury. The research participants of the literature included in this paper are all pre-professional dancers from dance school, with similar educational backgrounds and ages. Therefore, the years of receiving professional dance training and training load are similar. In addition, the physical health conditions of the same age group, such as bone density and muscle strength, or the development of physical qualities, such as agility and coordination, are generally the same, so there is no difference in injuries caused by age factors. In the literature confirming the relationship between age and dance injury, the results of different studies are contradictory. Some studies (26) have shown that a higher age is associated with the generation of contemporary dance injury. On the contrary, other works of literature suggest that the younger the professional dancers are, the more likely they are to be injured. In addition, studies on ballet and modern dance (5) have not found a correlation between age and the occurrence of serious injuries. Among the research studies, there are various types of dances that have multiple training methods. As a result, dancers of the same age produce injuries by different mechanisms. The research results of this paper also suggest that when exploring the influence of age factors on dance injury, research participants of different ages or obvious age differences should be selected, which will make the results more accurate and reliable.

This paper shows that there is a high correlation between BMI and injury, so high BMI is a risk factor for dancers to be injured. Some works of literature show that the possibility of injury will increase by 0.38 times with every higher point of BMI, and the higher the BMI (8), the higher the incidence of injury, but some studies believe that low BMI is the cause of injury (8). Both conclusions seem convincing. A low BMI indicates a slim body, without the protection of fat, and it can be easier for dancers to fracture. A high level of BMI may cause a decreased alertness which could increase the rate of falling. The difference in the research can be solved by unifying the dance type and training mode of participants. BMI is an index to evaluate the degree of body fat and thinness, and a high BMI makes dancers bear more load during training and performance (19). The participants included in this study are all undergraduate students. Obesity and overweight caused by physical development or diet and rest will increase the load on joints, ligaments, and other structures, while the high dance exposure hours will aggravate the generation of dance injuries. Therefore, the results of this study suggest that dancers should have a normal BMI not only to enhance the beauty and ornamental value of the dance but also more importantly to ensure the health of dancers and reduce injuries. The results of this study also suggest that in order to maintain the pertinence of the research, the specific BMI cut-off point should be divided according to the different ages and dance types.

The results of this study confirm that injury history is highly correlated with re-injury in contemporary dancers. All the participants in this paper have received professional dance training, so they have suffered various types and degrees of injury. With the increase in grades and the difficult setting of school courses, the intensity of training and the density of rehearsal and performance also increase, and the probability of re-injury is greater. Dancers do not receive active treatment and adequate rest after injury and participate in high-intensity training and performances before the old injury is fully recovered, thus causing new injuries, which is considered to be the mechanism of injury. In the injury studies of ballet and modern dancers, it was also found that the incidence of new injury was significantly higher among dancers with previous injury experience, reaching 63 and 35%, respectively (19). The results suggest that unhealed old injuries can continue to affect dancers, causing them to repeat injuries in subsequent training or create new injuries in other parts of the body.

As a risk factor, educational program was not shown to be a risk factor in this study, which is consistent with the conclusion of other studies. In other related studies, such as on professional or amateur dancers, this factor is not included. At present, only one study on ankle joints has found that the injury of students majoring in dance education is more serious than that of students majoring in dance performance (6). In this study, it is found that the heterogeneity of the injury factor of undergraduate majors is moderate, which may be related to the training programs, curriculum arrangements, and training methods of students in different majors. This result also suggests that we should try our best to select research participants under the same curriculum system or training mode when conducting injury difference analysis among students of different majors so that the research results will not be interfered with by other factors.

In this study, evidence-based medicine was used for the first time in the field of dance to conduct a meta-analysis of injury risk factors and prevalence in modern and contemporary dance. The results of this study not only point out the risk factors of contemporary dance injuries but also provide guidance for the prevention of future dance injuries.

### Limitation

In recent years, however, there have been few published prospective studies on modern and contemporary dance injury, and there are some problems such as missing data and small sample size in studies, which make it very difficult to collect, utilize, and analyze data comprehensively. There are many influencing factors leading to dance injury, and the factors included in the current research are limited, which makes the research results lacking in comparison and test. At present, the injury scale for dance mainly refers to the physical education participants, which lacks pertinence and scientific rationality for dance majors. In this study, the small sample sizes for subgroup analyses may also cause high heterogeneity. Therefore, in future studies, researchers should expand the scope, quantity, and professional identity of research participants, as well as follow the quality requirements of methodology and strictly control the occurrence of all kinds of bias so as to comprehensively improve the quality of published literature. In addition, scholars should expand the study of influencing factors, comprehensively consider the potential causes of injury, and prevent the occurrence of injury from all aspects. In the future, researchers should form an independent research system of dance specialty after conducting research on multiple dances, groups, and methods.

## Conclusion

This study collected foreign research on the influencing factors of dance injury in 20 years, taking pre-professional and professional dancers as the research participants. This paper systematically and comprehensively summarizes the correlation strength between five different factors and the risk of injury, and among them, injury history and high BMI are risk factors for dancers. From the analysis of prevalence, it can be seen that musculoskeletal injury is general among dancers, and the proportion of overuse and lower extremities is very high. Therefore, modern and contemporary dancers should maintain proper weight, reduce and prevent injury, and do dance training after the recovery of an injury so as to reduce the adverse effects. In future research, more details about race, training mode, and dance school can be added to the analysis of prospective studies which can increase the risk factors for dancing injury. After further research on the risk factors and injury mechanism, it seems possible to form specific injury prevention methods for modern and contemporary dancers.

## Author contributions

YS: Conceptualization, Data curation, Formal Analysis, Investigation, Methodology, Software, Writing – original draft. HL: Project administration, Supervision, Writing – review & editing.
