# Supplementary material for: Prevalence and risk factors of musculoskeletal injuries in modern and contemporary dancers: a systematic review and meta-analysis
Source: Front Public Health. 2024 Feb 28;12:1325536. doi: 10.3389/fpubh.2024.1325536 (PMC10932947; doi:10.3389/fpubh.2024.1325536)
Supplement: Supplementary file 1 [file Data_Sheet_1.PDF]

Table 1 Basic information of the included literature

Publication year<br>Study<br>Country	Follow up and months/weeks of training year	Age (years) [mean±SD]	Number (F/M)	Genre	Level	Injury definition
2003<br>Bronner et al[10]<br>America	5 prospective training years<br>195 weeks	24±2.6	42<br>（21/21）	Modern	Professional dancers	Any musculoskeletal complaint resulting in finical outlay.<br>(overuse, trauma)
2011<br>Ojofeitimi & Bronner[11]<br>America	8 prospective training years<br>328 weeks	27.3±0.3	87	Modern	Professional dancers	Any musculoskeletal complaint resulting in finical outlay.<br>(time-loss injury,overuse,trauma)
2012<br>Selina et al[13]<br>America	1 prospective training year<br>24 weeks	30.1±7.3	184<br>(135/49）	Modern	Professional dancers	Medical problems occurred as a result of participation in class, rehearsal,performance
2016<br>Bronner &Wood[22]<br>America	1 prospective training year<br>23 weeks	29.06±5.57	35<br>（18/17）	Modern	Professional dancers	WMSI(find the physical therapist)<br>Time-loss injury(cannot dance for several days)<br>Complaints(not TLinj or WMSI,including MAC and TCBC)
2016<br>Jacobs et al[4]<br>Canada	1 prospective training year<br>41 weeks	25±4.0	82	Ballet And modern	Professional dancers	Self-reported injury<br>Self-estimated functional inability because of pain

2017 van Seters et al[7] Netherlands	1 prospective training year 10 months	18.6±1.1	45 （28/17）	Contemporary	Pre - professional dancers	Self-reported injury (use OSTRC: Questionnaire on Health Problems)
2017 Kenny et al[14] Canada	1cross-sectional training year	23.5±6.5	65 （63/2）	Ballet and contem -porary	Pre-professional dancers	Self-reported injury(a baseline questionnaire)
2018 Bronner & Bauer[15] America	4 prospective training years 136 weeks	18.2±0.7	180	Modern	Pre-professional dancers	Self-reported injury(a questionnaire and the same physical therapists)
2018 Cahalan et al[17] Ireland	1 prospective training year 48 weeks	21.0±3.1	29 （28/1）	Contem-porary	Pre-professional dancers	Self-reported injury(a questionnaire
2018 Bronner et al[15] America	15 prospective training years 555weeks	25.2±0.8	159	Modern	Professional dancers	Time-loss injury Medical-attention injury All-complaints injury
2018 Kenny et al[16] Canada	1 prospective training year 10 months	17.9±6.6	60 （58/2）	Ballet and contem-porary	Pre-professional dancers	Work-related musculoskeletal injuries(WMSI) Time-loss injury
2019 van Winden[24] Netherlands	1 prospective training year 10 months	19.4±1.5	134 （90/44）	Contem-porary	Pre-professional dancers	Self-reported injury (use OSTRC: Questionnaire on Health Problems)
2020 Jeffries et al[25] Australia	1 prospective training year	26.1±5.1	16 （9/7）	Contem-porary	Professional dancers	Medical-attention injury Time-loss injury

2020 van Winden et al[8] Netherlands	1 prospective training year 10 months	19.2±1.5	99 （71/28）	Contem-porary	Pre-professional dancers	Self-reported injury (use OSTRC: Questionnaire on Health Problems)
2021 van Rijn et al[9] Netherlands	1 prospective training year 10 months	19.1±1.3	195 （127/68）	Contem-porary	Pre-professional dancers	Self-reported injury (use OSTRC: Questionnaire on Health Problems)
2021 van Winden et al[26] Netherlands	1 prospective training year 10 months	22.8±5.8	186 （127/59）	Contem-porary	Pre-professional dancers	Self-reported injury (use OSTRC: Questionnaire on Health Problems)
2021 Bronner et al[18] America	4 prospective training years 40 months	18.2±0.7	180 （140/40）	Modern	Pre-professional dancers	Medical attention injury(MAI) Time-loss injury(TLI)
2021 Adinda K et al[6] Netherlands	1 prospective training year 10 months	19.2±1.5	91 （64/27）	Contem-porary	Pre-professional dancers	Self-reported injury (use OSTRC: Questionnaire on Health Problems)
